# The deubiquitinase USP13 stabilizes the anti-inflammatory receptor IL-1R8/Sigirr to suppress lung inflammation

**DOI:** 10.1016/j.ebiom.2019.06.011

**Published:** 2019-06-14

**Authors:** Lian Li, Jianxin Wei, Shuang Li, Anastasia M. Jacko, Nathaniel M. Weathington, Rama K. Mallampalli, Jing Zhao, Yutong Zhao

**Affiliations:** a Department of Physiology and Cell Biology, Davis Heart and Lung Research Institute, The Ohio State University, Columbus, OH, USA; b Department of Respiration Medicine, Tianjin Medical University General Hospital, Tianjin, China; c Department of Surgery, The first affiliated hospital of Dalian Medical University, Dalian, China; d Department of Medicine, University of Pittsburgh School of Medicine, Pittsburgh, PA, USA; e Department of Internal Medicine, The Ohio State University, Columbus, OH, USA

**Keywords:** Deubiquitinating enzyme, Anti-inflammation, Cytokine receptor, Protein stability

## Abstract

**Background:**

The Single immunoglobin interleukin-1 (IL-1)-related receptor (Sigirr), also known as IL-1R8, has been shown to exhibit broad anti-inflammatory effects against inflammatory diseases including acute lung injury, while molecular regulation of IL-1R8/Sigirr protein stability has not been reported. This study is designed to determine whether stabilization of IL-1R8/Sigirr by a deubiquitinating enzyme (DUB) is sufficient to suppress inflammatory responses and lessen lung inflammation.

**Methods:**

A molecular signature of ubiquitination and degradation of IL-1R8/Sigirr was determined using a receptor ligation chase model. The anti-inflammatory effects on USP13 were investigated. USP13 knockout mice were evaluated for stabilization of IL-1R8/Sigirr and disease phenotype in an acute lung injury model.

**Findings:**

IL-1R8/Sigirr degradation is mediated by the ubiquitin-proteasome system, through site-specific ubiquitination. This effect was antagonized by the DUB USP13. USP13 levels correlate directly with IL-1R8/Sigirr, and both proteins were reduced in cells and tissue from mice subjected to inflammatory injury by the TLR4 agonist lipopolysaccharide (LPS). Knockdown of USP13 in cells increased IL-1R8/Sigirr poly-ubiquitination and reduced its stability, which enhanced LPS-induced TLR4 signaling and cytokine release. Likewise, USP13-deficient mice were highly susceptible to LPS or *Pseudomonas aeruginosa* models of inflammatory lung injury. IL-1R8/Sigirr overexpression in cells or by pulmonary viral transduction attenuated the inflammatory phenotype conferred by the *USP13*^*−/−*^ genotype.

**Interpretation:**

Stabilization of IL-1R8/Sigirr by USP13 describes a novel anti-inflammatory pathway in diseases that could provide a new strategy to modulate immune activation.

**Fund:**

This study was supported by the US National Institutes of Health (R01HL131665, HL136294 to Y.Z., R01 GM115389 to J.Z.).

**Research in context**

**Evidence before this study**

While Sigirr has been known as an anti-inflammatory receptor against endotoxin-induced pro-inflammatory responses, molecular regulation of Sigirr protein stability has not been investigated.

**Added value of this study**

In the present study, we demonstrate that Sigirr protein stability is regulated by the ubiquitin-proteasome system. USP13 was identified as a deubiquitinating enzyme responsible for stabilizing Sigirr; thus USP13 exhibits an anti-inflammatory property. Stabilization of Sigirr by USP13 suppresses lung inflammation induced by LPS or bacteria.

**Implications of all the available evidence**

This study reveals a novel mechanism by which USP13 stabilizes the anti-inflammatory receptor Sigirr. Thus promoting Sigirr stability and/or USP13 activity may play a protective role in mitigating lung inflammation.

Alt-text: Unlabelled Box

## 1 Introduction

Sigirr (known as IL-1R8) belongs to the IL-1R superfamily [1]. Distinct from classic IL-1R family members, Sigirr consists of only a single extracellular Ig-like domain, a long cytoplasmic tail, and an intracellular Toll/IL-1R (TIR) domain [1]. Sigirr negatively regulates IL-1 and TLR signaling [[1], [2], [3], [4], [5], [6], [7]] and broadly dampens inflammatory responses during acute lung injury [8], brain inflammation [[9], [10], [11]], arthritis [12], colitis [2,13], and pulmonary tuberculosis [14]. Though IL-37 has been identified as a Sigirr ligand [15], the anti-inflammatory effect of Sigirr seems to be independent of ligation by IL-37 [8]. While Sigirr gene expression has been shown to be regulated by the transcriptional factor Sp1 [16], the molecular regulation of Sigirr protein stability has not been reported. Understanding the regulation of Sigirr protein turnover could provide a mechanistic basis for the modulation of Sigirr-mediated anti-inflammatory signaling.

Protein turnover is regulated by post-translational modification such as mono- or poly-ubiquitination [17]. The attachment of polyubiquitin chains to substrate proteins serves as a molecular tag for their degradation. Polyubiquitination is characterized as linear based on the type of ubiquitin chain linkage. Generally, Lysine 48 (K48) -linked ubiquitination leads to protein degradation in the proteasome [18]. Deubiquitinating enzymes (DUBs) remove ubiquitin chains from substrate proteins and often rescue them from degradation [19]. Accumulating evidence suggests that DUBs are critical modulators of inflammatory responses [20], and several DUBs such as A20, BRCC3, and STAMBP/AMSH among others regulate signaling pathways downstream of IL-1 family or TLR signaling [[21], [22], [23]]. The regulation of Sigirr's protein stability by either ubiquitin addition or DUB activity is not yet described.

In this report, we utilized a ligand-induced receptor degradation system to investigate Sigirr stability. Sigirr is site-specifically polyubiquitinated by K48 ubiquitin chains and degraded in the proteasome. We also demonstrate that the DUB USP13 stabilizes Sigirr to dampen inflammatory responses. USP13 is sufficient at suppressing inflammation during LPS and *Pseudomonas aeruginosa-*induced lung inflammation in mice. These studies further establish the importance of Sigirr in lung inflammation and provide a molecular mechanism for Sigirr stabilization during lung inflammation.

## 2 Materials and methods

### 2.1 Generation of *USP13* deficient mice

The *Usp13*^*−/−*^ mice were generated by the CRISPR/Cas9 system at the University of Pittsburgh. Exon 6 and Intron 18 of *Usp13* (chromosome 3 between position 32,865,806 and 32,917,828) were deleted. Only the *Usp13* gene is localized in the position on chromosome 3 (https://www.ncbi.nlm.nih.gov/genome/gdv/browser/?context=genome&acc=GCF_000001635.26). In brief, Cas9 mRNA and two sgRNA were injected into the fertilized embryos, and then embryos in 2-cell stages were transferred to oviducts of pseudopregnant female mice. The RNA sequence guides are GTGTGCCCGATGTGACCTGC and TCGAGGTGGACTTATGCACA. The potential founder F0 mice were genotyped based on genomic DNA isolated from mouse tails by PCR with the following primer sets: F52: CTAGGTGGTCCTGGGCTTTG, R52: CAGGCTCATGAGTCACCACA, and R31: ACTCACTATGGCCTCAGCAA. A 481 bp or an approximately 600 bp fragment was produced from the WT allele or the null allele, respectively. Chimeric offspring were crossed with C57BL/6 to generate *Usp13*^*+/−*^ mice. The F1 *Usp13*^*+/−*^ mice were further crossed with C57BL/6 background for at least 6 generations before use. *Usp13*^*+/−*^ mice identified by genotyping through PCR were intercrossed for the generation of *Usp13*^*−/−*^ mice. Sex-matched *Usp13*^*+/+*^ and *Usp13*^*−/−*^ littermates at 8–10 weeks were used for animal studies.

### 2.2 LPS- or *P. aeruginosa*-induced murine models of lung injury

All mice were housed in the specific pathogen-free animal care facility at the University of Pittsburgh and the Ohio State University in accordance with institutional guidelines and guidelines of the US National Institutes of Health. All animal experiments were approved by an institutional animal care and use committee (IACUC) at the University of Pittsburgh and the Ohio State University Animal Resources Centers. Lung inflammation models were performed by intratracheal administration of LPS or *P. aeruginosa*. Briefly, mice were given intratracheal administration of LPS (1 mg per kg body weight) or *P. aeruginosa* (strain PA103; 1 × 10^4^ colony-forming units per mouse). At designated time points after LPS or PA103 challenge, the mice were anesthetized before myocardial perfusions were performed with PBS *via* the right ventricle until lungs were cleared of blood, and then lungs were harvested for further analyses. For BAL collection, the lungs were lavaged two times with 1 ml sterile PBS at room temperature. The cell-free supernatants were harvested for ELISA assay after centrifuging at 1000 rpm for 5 min. The cell pellets were diluted in 1 ml sterile PBS, and the cells were counted with a hemocytometer. Cytospin preparations of BAL cells were stained with hematoxylin and eosin and viewed under light microscopy for inflammatory cell differential. For lentiviral vector delivery system, cDNA encoding human *Sigirr* was inserted into the pLVX-IRES-tdTomato vector (Clontech, Palo Alto, CA, USA); lentiviral vectors encoding Sigirr and their controls were generated with a lentivirus packaging system (Clontech, Palo Alto, CA, USA). C57/BL6 mice were given 50 μl lentivirus vectors (2 × 10^7^ plaque-forming units per mouse) *via* intratracheal administration for 5 d before intratracheal challenge with LPS or PA103 (doses described above).

### 2.3 H&E staining and immunohistochemistry

The left lungs from animals were inflated with 0.5 ml of 2% PFA after clearing of blood. The lung tissues were then fixed overnight, embedded in paraffin. The sections (5 μm thick) were cut and used for staining with hematoxylin and eosin to assess the degree of lung injury. Immunohistochemistry was performed as described below. In brief, sections were deparaffinized and rehydrated through graded alcohol. Antigen retrieval was performed by high-pressure heating with citrate buffer (Thermo Scientific, Fremont, CA, USA), then tissues were incubated with different antibodies at 4 °C overnight and HRP-polymer secondary antibodies (Santa Cruz Biotechnology, Santa Cruz, CA, USA) for 15 min and then incubated and developed using DAB solution (Santa Cruz Biotechnology, Santa Cruz, CA, USA). The antibody specific for USP13 or IL-1β was used for staining. Images were captured by EVOS inverted microscope.

### 2.4 Cells and reagents

Mouse lung epithelial cells (MLE12, SV40-immortalized mouse alveolar cell line with epithelial characteristics), RAW 264.7 cells (mouse macrophage-like virus-induced leukemia cell line), and BEAS-2B (human lung epithelial cell line) were obtained from American Type Culture Collection (ATCC, Manassas, VA). Human macrophage-like cells (monocyte-derived macrophages differentiated *in vitro* with M-CSF and IL-4) were purchased from StemCell Technologies (Vancouver, BC, Canada). Human TLR4 stable transfected HEK293 (HEK293/TLR4) cells were from InvivoGen (San Diego, CA, USA). Recombinant human IL-37 (a truncated form, Lys27-Asp192) was from R&D Systems (Minneapolis, MN, USA). Cycloheximide, leupeptin, and LPS were from Sigma Aldrich (St Louis, MO, USA). MG-132 was from EMD Chemicals (Gibbstown, NJ, USA). Immobilized protein A/G beads and control IgG, anti-Sigirr (for mouse, rat, and human origin), anti-phospho-JNK1, anti-JNK1, anti-NF-κB p65, anti-GAPDH, anti-IL-1β, and anti-Lamin A/C and anti-TLR4 were from Santa Cruz Biotechnology (Santa Cruz, CA, USA). Anti-USP13 and anti-I-κB were purchased from ProteinTech (Chicago, IL, USA). Anti-USP13 was purchased from Bethyl Laboratories (Montgomery, TX, USA). Antibodies against phospho-p38, p38, phospho-I-κB, HA tag, K48 ubiquitin, K63 ubiquitin, and ubiquitin were from Cell Signaling (Beverly, MA, USA). Anti-V5, mammalian expression plasmid pcDNA3.1/His-V5-topo, *Escherichia coli* Top10 competent cells and lipofectamine RNAi MAX reagent were purchased from Life Technologies (Gaithersburg, Maryland, USA). Superfect transfection reagent was from QIAGEN (Valencia, CA, USA). LipoJet™ reagent and GeneMute siRNA transfection reagent were from SignaGen (Ijamsville, MD, USA). Anti-IL18R1, human *Usp13* siRNA and control siRNA were purchased from Thermo Fisher Scientific (San Jose, CA, USA). Horseradish peroxidase-conjugated goat anti-rabbit and anti-mouse secondary antibodies were obtained from Bio-Rad Laboratories (Hercules, CA, USA). All commercially available materials used were of the highest quality.

### 2.5 Plasmid and siRNA transfection

The cDNA encoding human *Usp13* or human *Sigirr* and its truncations were inserted into pCDNA3.1/V5-His-Topo vector (Invitrogen, Carlsbad, CA, USA). The sequences of specific primer pairs are described below: *Sigirr* forward, CACCATGCCAGGTGTCTGTGATA, *Sigirr* reverse, AATATCATCCTTGGACACCA, *Sigirr* (1–160) reverse, GTTTATCTCCACCTCCCCAT, *Sigirr* (1–290) reverse, GGGCCTCCAGAGCAGCAAGG, *Sigirr* (1–374) reverse, CCCACTGGTGTGCGGTGGAG, *Usp13* forward, CACCATGATGCAGCGCCGGGGCGCCCT, *Usp13* reverse, GCTTGGTATCCTGCGGTAAA. Usp13C345A mutant plasmid was carried out using QuikChange Lightning Site-Directed Mutagenesis Kit (Agilent Technologies, La Jolla, CA). The sequences of specific primer sets are described below: *Usp13C345A* forward, CAGAGCTGAGATAGCTGCTGTTGCCCA，*Usp13C345A* reverse, AACCTGGGCAACAGCAGCTATCTCAGC. Plasmid transfection in MLE12 cells was performed with Lonza electroporation according to the manufacturer's protocol. LipoJet™ reagent was used for transfection of plasmids into RAW 264.7 cells and HEK293/TLR4 cells according to manufacturer instructions (Life Technologies, Gaithersburg, Maryland, USA). Cellular transfection of siRNA in BEAS-2B cells was done with GeneMute siRNA transfection reagent system.

### 2.6 qRT-PCR analysis

RNA isolation and qRT-PCR analysis were performed as previously described [24]. The expression of *Usp13* and *Sigirr* was performed using iQ SYBR Green Supermix and the iCycler real-time PCR detection system (Bio-Rad, Hercules, CA, USA). *GAPDH* was used as an internal control. The sequences of specific primer pairs are described below: *hUsp13* forward, TGTCGCAAGGCTGTGTACTT, *hUsp13* reverse, CAGCGGCTCAGCAAAATCTG; *hSigirr* forward, GGAAGCTCTACGACGCCTAC, *hSigirr* reverse, TCTGGGGGTCTCCTTCCAC; h*GAPDH* forward, TCGGAGTCAACGGATTTGGTCG, h*GAPDH* reverse, GCTCTCCAGAACATCATCCCTGCCT-3; *mUsp13* forward, AGTGCTCAGCTCAAAGTCCC, *mUsp13* reverse, AGTTGCACAAGGTTGTTGGC; *mSigirr* forward, CAGTGTCCTGGTGCTCAACT, *mSigirr* reverse, GCTCGCCAAAGAGTGAAGGA; m*Gapdh* forward, ACCCTTAAGAGGGATGCTGC, m*Gapdh* reverse, TCACACCGACCTTCACCATTT.

### 2.7 Immunostaining

MLE12 cells were grown in glass bottom dishes until 80–90% confluence. After treatment or transfection, cells were fixed with 3.7% formaldehyde for 20 min, blocked with 1% BSA in TBST for 30 min, then immunostained with HA tag or V5 tag antibody for 1 h followed by washes with PBS for three times, and incubated with the fluorescent probe-conjugated secondary antibodies. Images were captured by a Nikon ECLIPSE TE 300 inverted microscope.

### 2.8 Western blotting analysis

The proteins for cells or lung tissues were extracted using cell lysis buffer following standard protocols as described before [25]. The cell lysates were then sonicated on ice for 12 s, followed by centrifugation at 4 °C at 5000 rpm for 10 min to remove insoluble fragments. Protein concentrations were determined with a Bio-Rad Protein Assay Kit (Bio-Rad Laboratories, Inc., Hercules, CA, USA). Equal amounts of samples were subjected to SDS-PAGE gel. Blots were washed with 25 mM Tris HCl (pH 7.4), 137 mM NaCl, and 0.1% Tween 20 (TBST) and incubated with a primary antibody for 2 h or overnight. The membranes were then washed three times at 10 min intervals with TBST prior to the addition of a secondary antibody for 1 h. Blots were developed with an Enhanced Chemiluminescence Detection Kit (ThermoFisher Scientific, Waltham, MA) according to the manufacturer's instructions. Western signaling was detected by Azure c600 imaging system.

### 2.9 *In vitro* translation of cDNA of human Sigirr wild-type and mutants

Sigirr-V5, Sigirr (1–290)-V5, Sigirr (1–374)-V5 and control vector was translated with a TnT *in vitro* translation system as directed by the manufacturer's instructions (Promega, Madison, WI, USA). Translated V5-tagged wild type and mutant human Sigirr were analyzed by immunoblots probing the V5-tag.

### 2.10 Co-immunoprecipitation and *in vivo* ubiquitination assay

For immunoprecipitation, equal amounts of protein were incubated with a specific primary antibody overnight at 4 °C, and co-immunoprecipitation was performed as described before [25]. MLE12 cells were washed with cold PBS and harvested in cell lysis buffer containing 20 mM Tris-HCl (pH 7.4), 150 mM NaCl, 2 mM EGTA, 5 mM β-glycerophosphate, 1 mM MgCl2, 1% Triton X-100, 1 mM sodium orthovanadate, 10 μg/ml protease inhibitors, 1 μg/ml aprotinin, 1 μg/ml leupeptin, and 1 μg/ml pepstatin. Equal amounts of protein were incubated with the indicated antibodies or control IgG. Antibodies against Sigirr in combination with protein A/G plus agarose were used to pull down overexpressed or endogenous USP13 in MLE12 lysate. For the *in vivo* ubiquitination assay, we performed a modified protocol under denaturing conditions. Cells were treated with or without MG-132 and leupeptin for 2 h before they were collected. The supernatant was removed after centrifuging at 1000 rpm for 5 min, followed by the addition of 1 μl of ubiquitin aldehyde and 1 μl of NEM. According to the size of the pellet, 50–80 μl of 2% SDS lysis buffer was added. The cells were then boiled at 100 °C for 10 min after sonication. And then, the samples were diluted with 500–800 μl of 1 × TBS. Regular IP procedure was performed as described in the previous section.

### 2.11 Nuclear protein isolation

Cell nuclear proteins were extracted with the EpiQuik Nuclear Extraction Kit (Epigentek). Protein concentration was determined by the Bio-Rad Protein Assay Kit (Bio-Rad Laboratories, Inc., Hercules, CA, USA).

### 2.12 Quantification and statistical analysis

Protein band intensities were quantified by ImageJ software (Image Processing and Analysis in Java; National Institutes of Health, Bethesda, MD, USA; http://imagej.nih.gov/). Comparisons between several groups were analyzed using one-way or two-way ANOVA. Data are expressed as mean ± SEM of triplicate samples from at least three independent experiments and values that were p < 0.05 were considered statistically significant.

### 2.13 Enzyme-linked immunosorbent assay (ELISA)

Levels of TNF-α, IL-1β, IL-6, and IL-8 proteins in the BAL and cell supernatant were measured with commercial ELISA kits (eBioscience, San Diego, CA, USA) according to the manufacturers' instructions.

## 3 Results

### 3.1 Sigirr is degraded in the proteasome

**Fig. 1:**
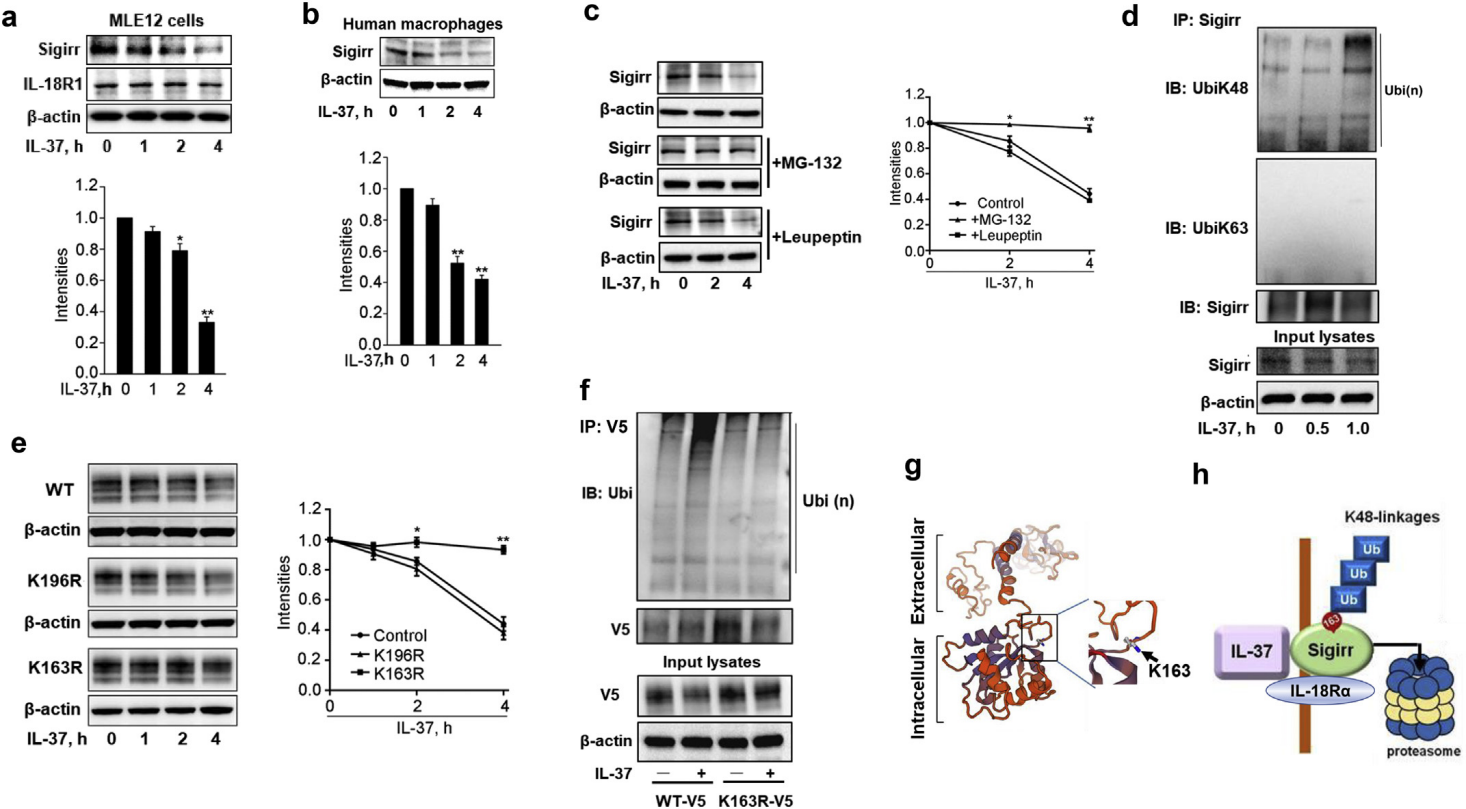
Sigirr degradation is mediated by the ubiquitin-proteasome system. (a, b) MLE12 (a) or human macrophage-like cells (b) were treated with IL-37 (100 ng/ml) for the indicated times with immunoblots to examine the protein levels of endogenous Sigirr, IL-18R1, and β-actin. (c) MLE12 cells were pretreated with MG-132 (20 μM) or leupeptin (100 μM) for 2 h and then treated with IL-37 (100 ng/ml) for the indicated times. (d) MLE12 cells were treated with IL-37 for the indicated times. *In vitro ubiquitination assay* was performed with a modified immunoprecipitation (IP). Smear bands indicate Ubi(n). (e) MLE12 cells were transfected with plasmids encoding Sigirr-V5, K196R-V5 mutant, or K163R-V5 mutant for 48 h and then treated with IL-37 for the indicated times. (f) MLE12 cells were transfected with plasmids encoding Sigirr-V5 and its mutant K163R-V5 for 48 h and then treated with IL-37 for 1 h. Denatured cell lysates were subjected to IP with a V5 antibody, followed by immunoblotting with an antibody against ubiquitin (Ubi). Smear bands indicate Ubi(n). (g) Predicated Sigirr protein structure analysis by Swiss-Model reveals that K163 is localized on the protein surface. (h) The scheme shows that IL-37 induces Sigirr degradation in the ubiquitin-proteasome system. Sigirr is polyubiquitinated by a K48-linked chain on the K163 residue. (For all panels, all data are mean ± SEM. n = 3 *p < 0.05; **p < 0.01 by one-way (a, b) or two-way (c, e) ANOVA with *Post hoc* Tukey's test compared to 0 h or control. Representative blots from three independent experiments are shown. Bands densitometry were analyzed with Image J.

Most cell surface receptors are post-translationally modified, internalized, and degraded upon ligation as a primary control for receptor abundance and intracellular signaling [[26], [27], [28]]. IL-37 has been identified as Sigirr's only ligand [1]. We tested if IL-37 modulates Sigirr abundance. Treatment of MLE12 mouse lung epithelial cells or human macrophage-like cells with IL-37 decreased total Sigirr protein levels but not IL-18R1 as assessed by immunoblotting (Fig. 1a, b). Flow cytometry analysis with an anti-Sigirr antibody revealed that IL-37 reduced its abundance on the cell surface over time (Supplemental Fig. S1a). IL-37 also significantly reduced overexpressed Sigirr-V5 in a dose-dependent manner (Supplemental Fig. S1b). Notably, Sigirr mRNA levels were not reduced by IL-37 in 2 h, while at 4 h, Sigirr mRNA level was significantly increased (Supplemental Fig. S1c), possibly as a compensatory response to reduced protein levels. These results indicate that Sigirr internalization and degradation by IL-37 is a suitable molecular model for investigating the molecular mechanisms of Sigirr degradation. To examine if the proteasome or lysosome pathways were involved in the degradation of Sigirr, we incubated MLE12 cells with inhibitors of the proteasome (MG-132) or lysosome (leupeptin) activity prior to IL-37 treatment. MG-132, but not leupeptin, prevented Sigirr degradation (Fig. 1c, Supplemental Fig. S1d). After IL-37 treatment, the transfected Sigirr-V5 protein product did not localize in the lysosomes in MLE12 cells (Supplemental Fig. S2a), suggesting that Sigirr degradation does not occur in the lysosome. In addition, the half-life of Sigirr (~4 h) in the presence of the protein synthesis inhibitor cycloheximide (CHX) was prolonged by MG-132, but not by leupeptin (Supplemental Fig. S2b), further implying that proteasomal degradation is the major pathway of cellular Sigirr elimination.

Polyubiquitination can be characterized by Lys (K)48-, K63-, and other linkages of ubiquitin chains based on which lysine residue of ubiquitin is bound to a growing ubiquitin chain [29]. Next, we examined Sigirr ubiquitination with a linkage-specific polyubiquitin pulldown and revealed that the addition of K48- and not K63-linked ubiquitin chain is the predominant mode of Sigirr modification (Fig. 1d). Ubiquitin chains are usually added to lysine residues on substrate proteins [30]. To identify putative ubiquitin-acceptor sites within Sigirr, we replaced several K candidate residues of human Sigirr with arginine (R). These K-to-R mutations identified the K163 residue of Sigirr as a putative ubiquitin acceptor residue for IL-37-mediated modification, as SigirrK163R was not degraded and displayed a reduction in ubiquitination compared to WT or other Sigirr K-to-R mutants (Fig. 1e, f). K163 seems to be localized in a surface-exposed loop in the intracellular domain of Sigirr (Fig. 1g). Notably, this ubiquitin site mutation of Sigirr (SigirrK163R) had no effect on ligand-induced Sigirr internalization (Supplemental Fig. S3a, b), suggesting that ubiquitination at K163 is not necessary for Sigirr internalization. These data suggest that Sigirr can be ubiquitinated by K48-linked ubiquitin chains on the K163 residue in response to IL-37 ligation, resulting in its proteasomal degradation (Fig. 1h).

### 3.2 USP13 deubiquitinates and stabilizes Sigirr

**Fig. 2:**
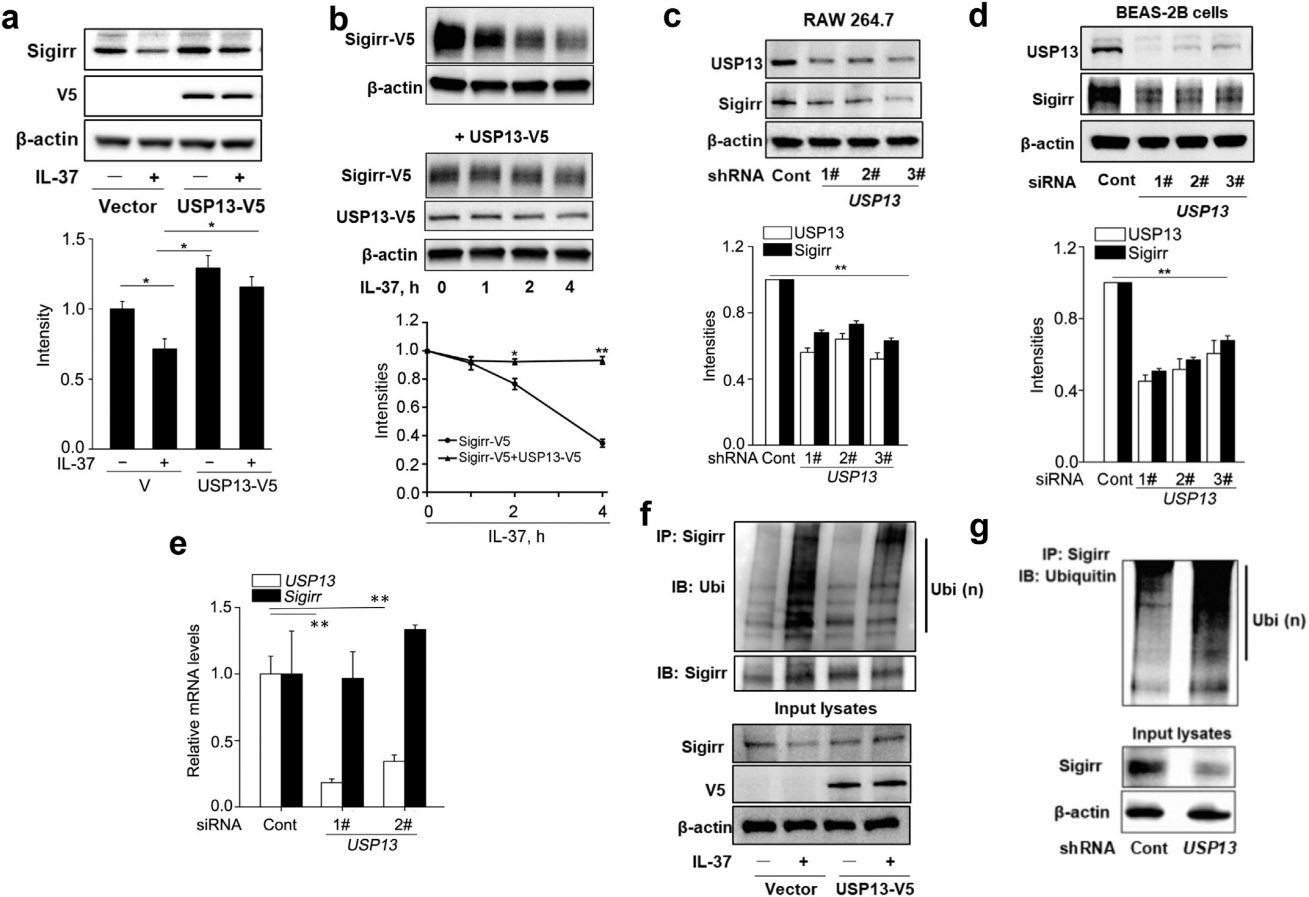
USP13 deubiquitinates and stabilizes Sigirr. (a) MLE12 cells were transfected with either an empty vector or USP13-V5 plasmid for 48 h and then treated with IL-37 for 2 h. Cell lysates were immunoblotted with Sigirr, V5 tag, and β-actin antibodies. (b) USP13-V5 plasmids were co-transfected with Sigirr-V5 plasmids into MLE12 cells and then treated with IL-37 for 0–4 h. RAW 264.7 cells were transfected with control shRNA or three *mUSP13* shRNAs (c), in BEAS-2B cells transfected with control siRNA or three *USP13* siRNAs (d). USP13 and Sigirr were evaluated by densitometric analysis. (e) BEAS-2B cells were transfected with control siRNA, or two *USP13* siRNAs for 3 d. qRT-PCR was performed to analyze the relative mRNA expressions of *Sigirr* and *USP13.* (f) MLE12 cells were transfected with plasmids encoding either an empty vector or USP13-V5 for 48 h and then treated with IL-37 for 2 h. Denatured cell lysates were immunoprecipitated with a Sigirr antibody, and the immunoprecipitated complexes were immunoblotted with an antibody against Ubi. Smear bands indicate Ubi(n). Input cell lysates were immunoblotted with Sigirr, V5 tag, and β-actin antibodies. (g) MLE12 cells were transfected with control shRNA or *USP13* shRNA for 72 h. Denatured cell lysates were immunoprecipitated with a Sigirr antibody, and the immunoprecipitated complexes were immunoblotted with an antibody against Ubi. Smear bands indicate Ubi (n). Input cell lysates were immunoblotted with Sigirr and β-actin antibodies. For all panels, all data are mean ± SEM. n = 3 *p < 0.05; **p < 0.01 by one-way (b, d, e) or two-way (a) ANOVA with *Post hoc* Tukey's test compared to control. Representative blots from three independent experiments are shown. Bands densitometry were analyzed with ImageJ.

Since DUBs remove ubiquitin chains, they can oppose degradation signals to stabilize substrate proteins. We performed a screen to identify a DUB responsible for stabilizing Sigirr (Supplemental Fig. S4). Only ectopic expression of V5-tagged USP13 (USP13-V5) significantly diminished degradation of endogenous or exogenous Sigirr (Fig. 2a, b). Three separate short hairpin RNAs (shRNAs) targeting mouse *USP13* mRNA effectively reduced Sigirr protein levels by ~70–90% (Fig. 2c), and likewise, human *USP13* siRNAs also decreased Sigirr protein by ~80% in BEAS-2B human epithelial cells (Fig. 2d). Knockdown of *USP13* did not affect *Sigirr* mRNA transcript levels (Fig. 2e), indicating that USP13 stabilizes Sigirr protein without altering its gene expression. Overexpression of USP13-V5 attenuated Sigirr ubiquitination in MLE12 cells (Fig. 2f), while knockdown of *USP13* with shRNA (Fig. 2g) enhanced Sigirr ubiquitination, suggesting that USP13 deubiquitinates Sigirr to stabilize the protein.

### 3.3 USP13 is associated with Sigirr

**Fig. 3:**
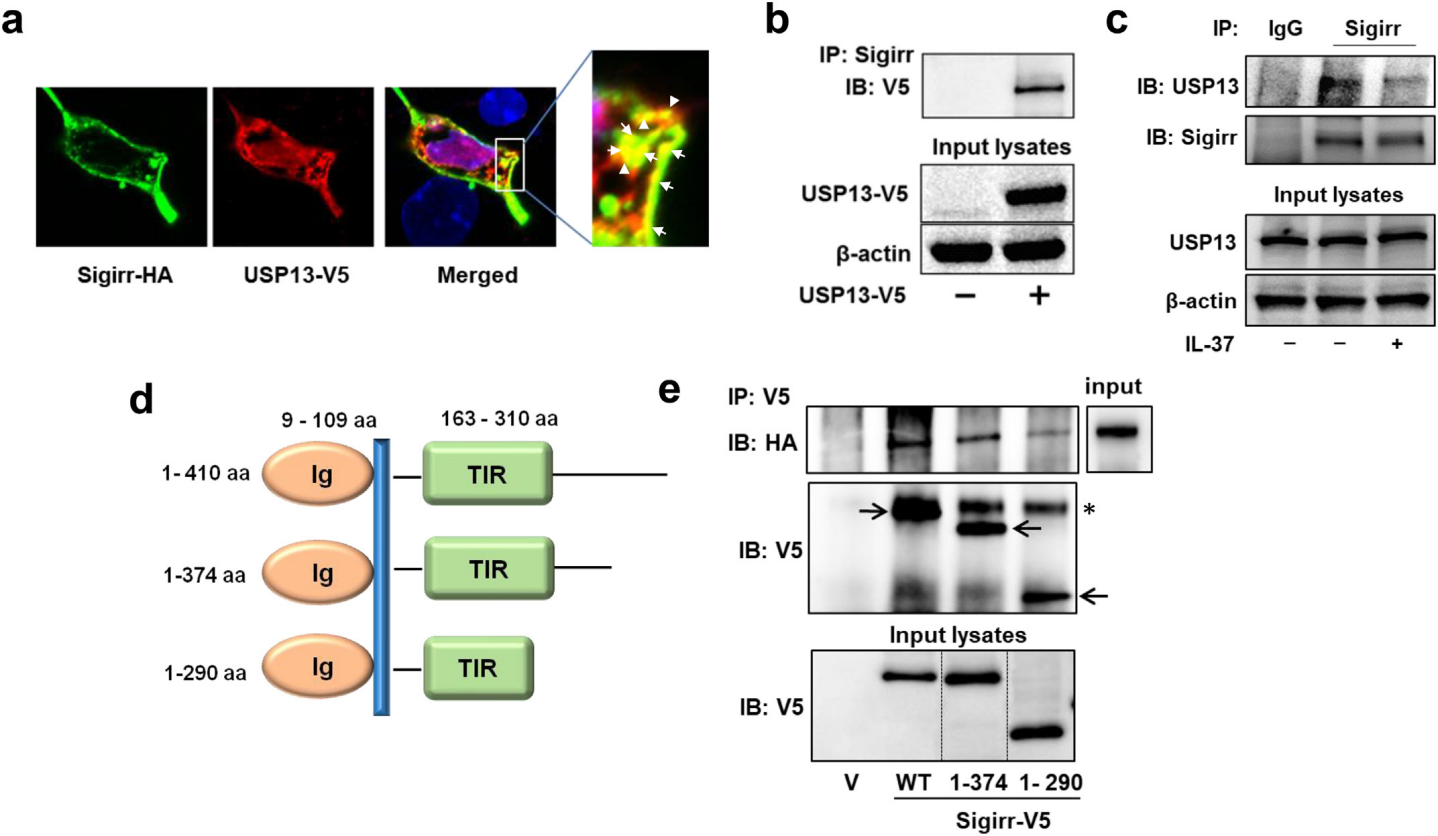
USP13 is associated with Sigirr. (a) Sigirr-HA and USP13-V5 co-overexpressed MLE12 cells were double-immunostained with V5 and HA antibodies. Scale bar, 5 μm. Yellow (shown by arrows) indicates co-localization. (b) Overexpressed USP13-V5 in MLE12 cells were co-immunoprecipitated with a Sigirr antibody and then analyzed by immunoblotting with a V5 antibody. Input cell lysates were immunoblotted with V5 and β-actin antibodies. (c) MLE12 cells were treated with or without IL-37 for 1 h. Cell lysates were assessed by IP with an antibody against Sigirr and immunoblot analysis of precipitates with antibodies against USP13 and Sigirr. Input lysates were analyzed with antibodies against USP13 and β-actin. (d) Schematic representation of V5-tagged full-length Sigirr (1–410 aa) and two C-terminal deletion mutants. Ig: immunoglobin; TIR: the Toll/IL-1R domain. (e) Control vector (V), USP13-HA, and V5-tagged Sigirr or its C-terminal deletion mutants were synthesized with a TnT *in vitro* translation system. USP13-HA was incubated with Sigirr-V5 or its mutants, followed by IP with anti-V5 beads and immunoblotted with an antibody to HA. Input lysates were analyzed by antibodies to HA or V5 tag. The arrows indicate overexpressed Sigirr-V5 and mutants. A star (*) indicates a non-specific band. For all panels, Representative blots or images from two (e) or three (all others) independent experiments are shown. (For interpretation of the references to colour in this figure legend, the reader is referred to the web version of this article.)

Next, we evaluated USP13-Sigirr association using co-immunofluorescence staining. MLE12 cells were co-transfected with HA-tagged Sigirr (Sigirr-HA) and USP13-V5. Sigirr-HA and USP13-V5 were highly co-localized on the inside of the cell membrane with some puncta also observed in the cytoplasm (Fig. 3a). Co-immunoprecipitation (Co-IP) also revealed USP13's association with Sigirr (Fig. 3b, c). The association between DUB and a transmembrane receptor protein can be regulated by ligation [28]. As shown in Fig. 3c, the complex of USP13 and Sigirr is disrupted after IL-37 treatment, indicating that IL-37 increases Sigirr ubiquitination through the reduction of its binding to USP13. To further identify the USP13-binding region on Sigirr, we incubated purified USP13-HA together with a series of C-terminal deletion mutants of Sigirr-V5 (Fig. 3d). The Co-IP assays revealed that the 290-374aa region in the C-terminal of Sigirr is required for its association with USP13 (Fig. 3e).

### 3.4 USP13 dampens LPS-induced inflammatory responses via the stabilization of Sigirr

**Fig. 4:**
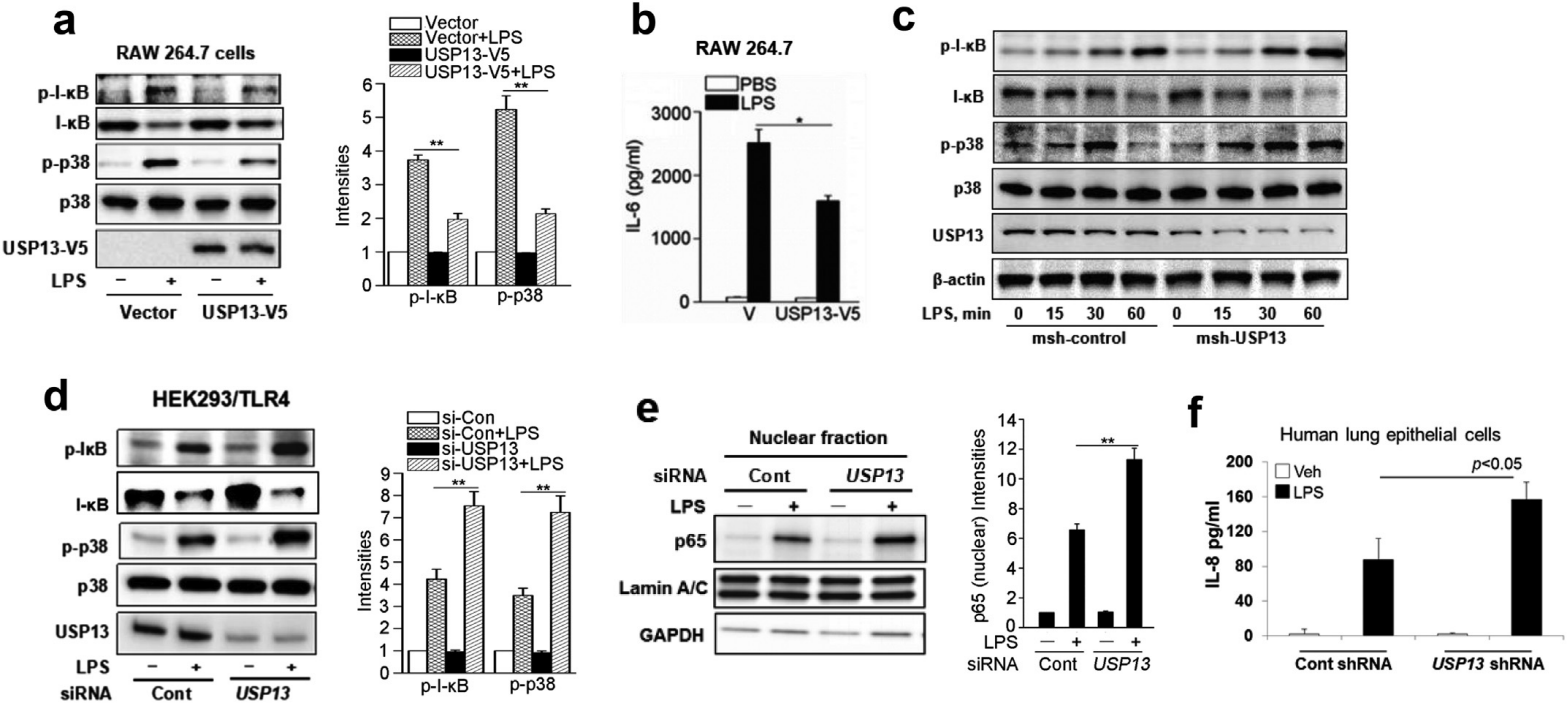
USP13 attenuates LPS-induced inflammatory responses. (a) RAW 264.7 cells were transfected with plasmids encoding either an empty control vector or USP13-V5 and then treated with LPS (0.1 μg/ml) for 30 min. Cell lysates were analyzed by immunoblotting with indicated antibodies. (b) MLE12 cells were transfected with plasmids encoding either a control vector or USP13-V5 and then treated with LPS (10 μg/ml) for 16 h. The supernatant was used to examine IL-6 release (c) RAW 264.7 cells were transfected with control siRNA, or *USP13* siRNA for 3 d and then treated with LPS (0.1 μg/ml) for 0, 15, 30, 60 min. Cell lysates were analyzed by immunoblotting with indicated antibodies. (d) HEK293/TLR4 cells were transfected with control siRNA, or *USP13* siRNA for 24 h and then transfected with plasmids encoding empty vector or Sigirr-V5 for an additional 48 h. The cells were treated with LPS (0.1 μg/ml) for 30 min, and cell lysates were analyzed by immunoblotting with indicated antibodies. (e) RAW 264.7 cells were transfected with control siRNA, or *USP13* siRNA for 72 h, and then cells were treated with LPS (0.1 μg/ml) for 30 min. Nuclear fractions were isolated, and nuclear lysates were analyzed by immunoblotting with NF-κB p65, Lamin A/C, and GAPDH. (f) Human bronchial epithelial cells were transfected with control siRNA or *USP13* siRNA for 72 h, and then cells were treated with or without LPS (5 μg/ml) for 6 h. Supernatants were collected to measure IL-8 by ELISA. For all panels, all data are mean ± SEM. n = 3 *p < 0.05; **p < 0.01 by two-way ANOVA with *Post hoc* Tukey's test compared to vector + LPS group (a, b) or sicontrol + LPS (d, e). Representative blots from three independent experiments are shown. Bands densitometry were analyzed with Image J.

To investigate the biological function of USP13 in inflammatory responses, we first confirmed the anti-inflammatory activity of Sigirr. Overexpression of Sigirr attenuated LPS-induced phosphorylation of JNK, ERK, and IL-6 gene expression (Supplemental Fig. S5a, b). We then modulated USP13 and observed downstream inflammatory signals in a cellular model. LPS induces NF-κB and MAPK signaling to mediate inflammatory signaling and cytokine release. Overexpression of USP13 attenuated LPS-induced phosphorylation and degradation of I-κB, phosphorylation of p38 (Fig. 4a), as well as LPS-induced IL-6 release in RAW 264.7 cells (Fig. 4b). Downregulation of *USP13* with shRNA or siRNA promoted LPS-induced phosphorylation and degradation of I-κB, activation of p38 (Fig. 4c, d), NF-κB p65 subunit nuclear translocation (Fig. 4e), and IL-8 release (Fig. 4f). To determine if the inhibitory effect of USP13 on LPS signaling is dependent on its activity, we overexpressed a catalytically inactive mutant of USP13 (C345A) in 293TLR4 cells. As shown in Supplemental Fig. S5c, USP13C345A did not attenuate LPS-induced phosphorylation of p38 but rather increased p38 phosphorylation, suggesting that the enzyme activity of USP13 is required for inhibiting LPS signaling. These data indicate that USP13 exhibits anti-inflammatory activity in LPS-exposed cells.

### 3.5 USP13 is reduced in response to endotoxin exposure in vitro and in vivo

**Fig. 5:**
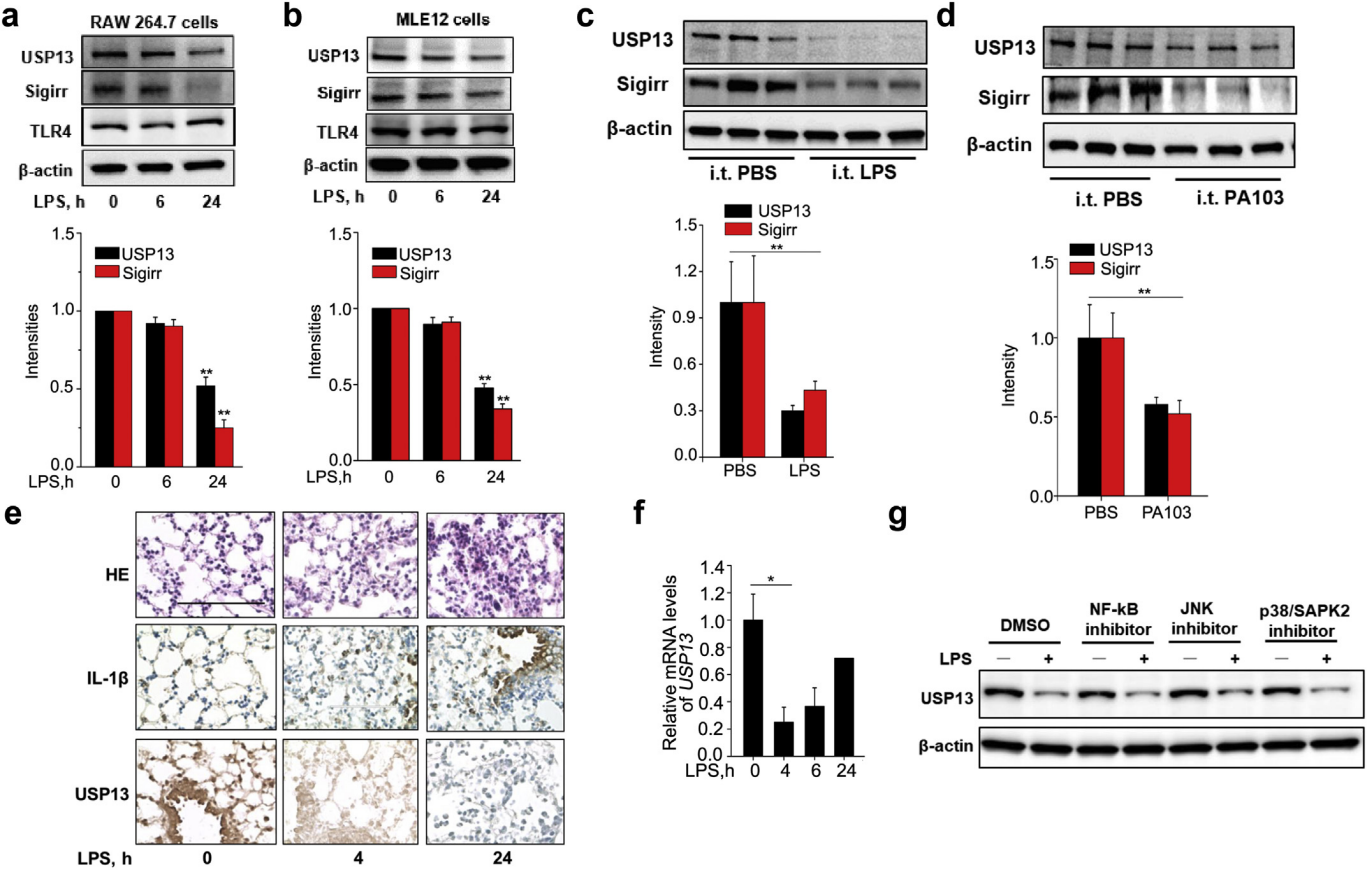
USP13 is reduced in response to endotoxin exposure. (a, b) RAW 264.7 (a) or MLE12 cells (b) were treated with LPS (0.1 μg/ml for RAW 264.7 cells; 5 μg/ml for MLE12 cells) for 0, 6, and 24 h. Cell lysates were analyzed by immunoblotting with indicated antibodies. (c, d) C57BL/6J mice were challenged with an intratracheal injection of LPS (1 mg/kg body weight, 24 h) (c) or *P. aeruginosa* (strain PA-103) for 4 h (d). Lung tissue lysates were analyzed by immunoblotting with antibodies against USP13, Sigirr, and β-actin. (e) Lung tissues were stained by hematoxylin and eosin (H&E) and antibodies against IL-1β or USP13. Representative images are shown (n = 4–6 per group). Bars, 100 μm. (f) RAW 264.7 cells were treated with LPS (0.1 μg/ml) for 0, 4, and 6 h. qRT-PCR was performed to analyze the relative mRNA expression of *USP13* (g) RAW 264.7 cells were treated with an NF-κB inhibitor (10 μM), JNK inhibitor (10 μM), or p38/SAP2 inhibitor (10 μM) for 1 h prior to LPS (0.1 μg/ml, 30 min) treatment. Cell lysates were analyzed by immunoblotting with antibodies against USP13 and β-actin. For all panels, all data are mean ± SEM. n = 3 *p < 0.05; **p < 0.01 by two-way ANOVA with *Post hoc* Tukey's test compared to 0 h (a, b). Representative blots from three independent experiments are shown. Bands densitometry were analyzed with ImageJ.

Following LPS exposure, USP13 and Sigirr, but not TLR4, were both depleted over time in RAW 264.7 cells and MLE12 cells (Fig. 5a, b). In murine models of acute lung injury, USP13 and Sigirr levels were likewise decreased in mice lung homogenates after intratracheal challenge with either LPS or *P. aeruginosa* (strain PA103) (Fig. 5c, d). Immunohistochemical staining from these experiments revealed reductions in USP13 in the lung epithelium, which correlated with increased inflammatory injury indicated by H&E staining and IL-1β abundance (Fig. 5e). These data suggest that USP13 is downregulated during acute inflammatory injury. We evaluated possible molecular mechanisms of LPS-induced USP13 downregulation and observed that *USP13* mRNA levels were reduced by LPS (Fig. 5f), suggesting that LPS stimulation diminishes USP13 transcription. We then tested several downstream pathways, and pharmacologic blockade of NF-κB, JNK, and p38 pathways did not affect the LPS-mediated reduction of USP13 (Fig. 5g). Further studies will be needed to determine if and how LPS might accelerate *USP13* mRNA degradation or cause epigenetic modification to the *USP13* promoter or promoter-related histone subunits (*e.g.*, methylation or acetylation).

### 3.6 Usp13 deficient mice exacerbate LPS or *Pseudomonas aeruginosa*-induced lung inflammation

To investigate the relevance of USP13 in the inflammatory response, we used CRISPR/Cas9 to generate *Usp13* deficient mice (Supplemental Fig. S6a, b). We noticed that there were faint bands close to the molecular weight of USP13 in lung tissue lysates from *Usp13*^*−/−*^ mice. It is possible that the faint bands are from non-specific detections or an undiscovered *Usp13* splicing mutant. *Usp13* deficient mice exhibit no significant phenotype differences compared to wild type mice in the normal condition. Consistent with our cellular USP13 knockdown studies (Fig. 2c, d), Sigirr levels in lungs from *Usp13*^*−/−*^ mice were significantly reduced (Supplemental Fig. S6b).

**Fig. 6:**
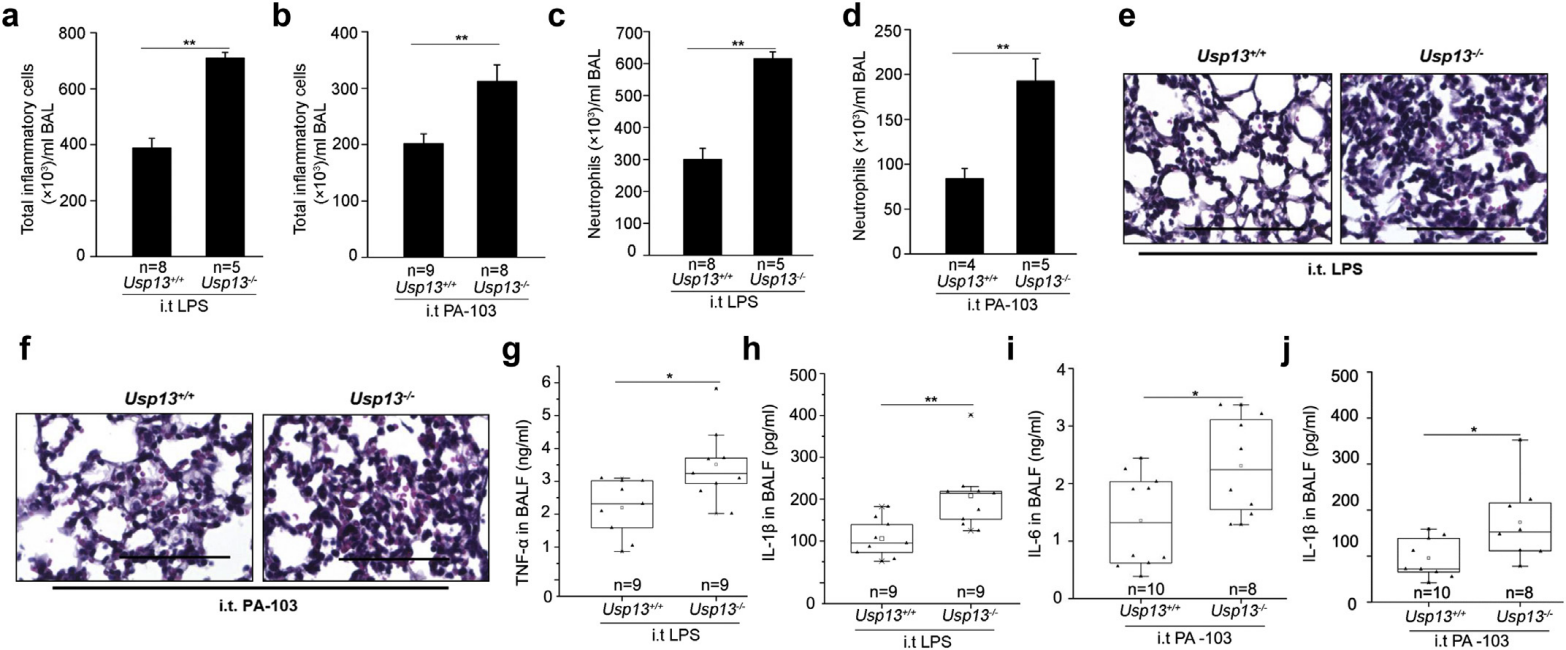
*Usp13*^*−/−*^ mice increase lung inflammation after LPS or PA103 challenge. WT and *Usp13*^*−/−*^ mice were subjected to intratracheal injection of LPS (1 mg/kg body weight) for 6 h (a, c, e, g, h) or *P. aeruginosa* (strain PA103; 1 × 10^4^ colony-forming units/mouse) for 4 h (b, d, f, i, j). (a, b) Total cell counts in BAL were measured by a hemocytometer. (c, d) Neutrophil-influx into alveolar spaces was examined by cytospin. (e, f) Lung sections were stained with hematoxylin and eosin. Representative images of the staining are shown (n = 8–10 per group). Bars, 100 μm. (g–j) TNF-α, IL-1β, and IL-6 levels in BAL were measured by ELISA and overall For all panels, the p-value was calculated by two-way ANOVA with *post hoc* Tukey's test *p < 0.05, **p < 0.01.

Based on the *in vitro* data, we hypothesized that *Usp13*^*−/−*^ mice would display enhanced inflammation, and we tested this with intratracheal administration of LPS or *Pseudomonas aeruginosa* (strain PA103) to *Usp13*^*−/−*^ or *Usp13*^*+/*+^ (WT) mice. Compared to WT mice, *Usp13*^*−/−*^ mice displayed a significantly greater cellular infiltration, particularly with neutrophils, 6 h after LPS or 4 h after *P. aeruginosa* challenge (Fig. 6a–d). *Usp13*^*−/−*^ mice also displayed increased inflammatory injury shown by H&E staining (Fig. 6e, f), and enhanced TNF-α, IL-1β, and IL-6 (Fig. 6g–j) release in BAL fluid. These data demonstrate that the deletion of USP13 promotes lung inflammation in murine models of lung injury. Taken together, this study indicates that the reduction of USP13 in the setting of inflammation may partly contribute to the pathogenesis of lung injury.

### 3.7 Sigirr reverses LPS-induced acute lung inflammation in Usp13^−/−^ mice

**Fig. 7:**
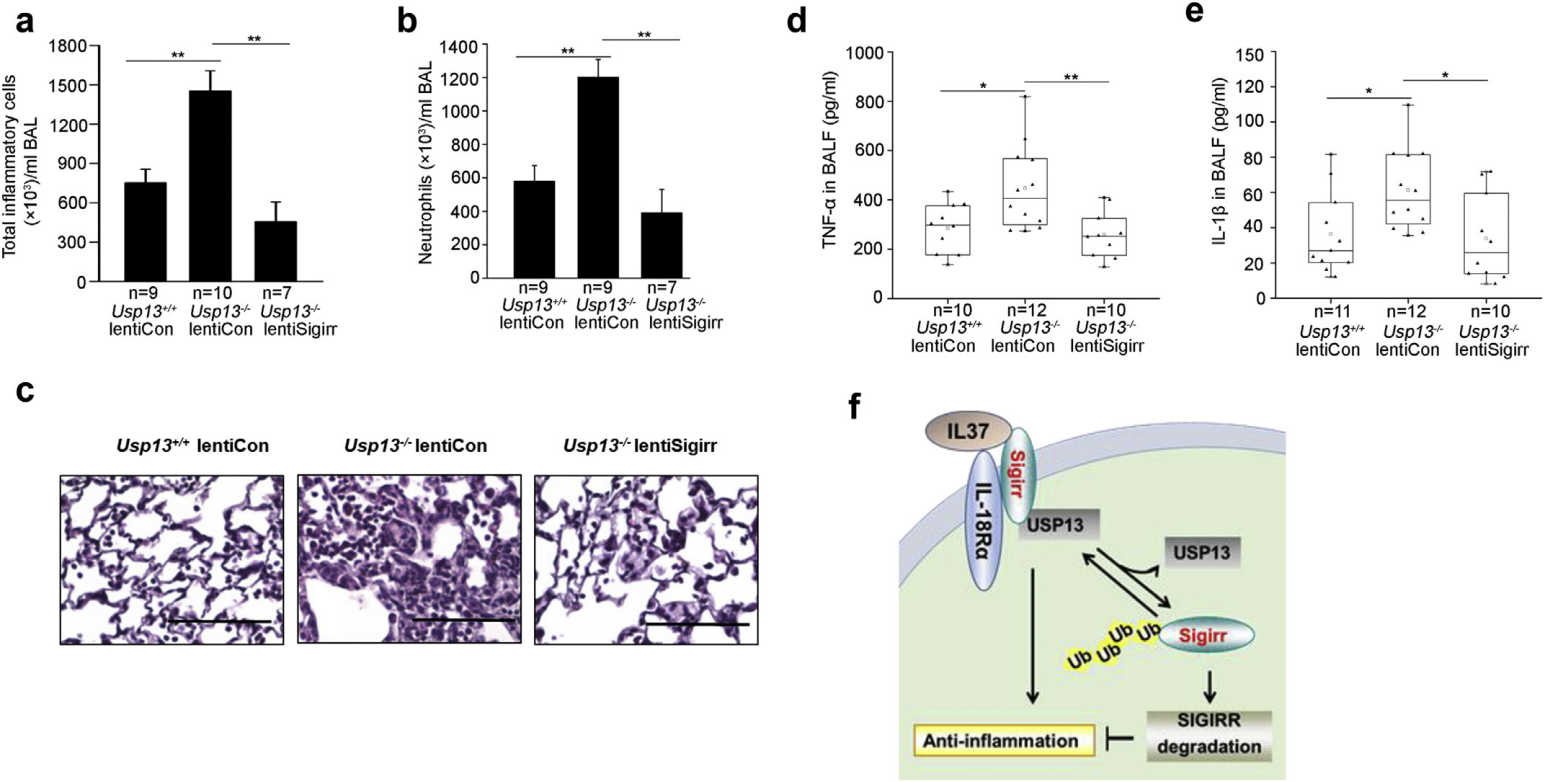
Sigirr reverses USP13 deficient-promoted lung inflammation. WT and *Usp13*^*−/−*^ mice were subjected to intratracheal injection of control lentivirus (LentiCon) or lentivirus encoding Sigirr (lentiSigirr) for 5 d and then intratracheal administration of LPS (2 mg per kg body weight) for 24 h. (a) Total cell counts in BAL. (b) Neutrophil-influx into alveolar spaces was examined by cytospin (c) Lung sections were stained with hematoxylin and eosin. Representative images of the staining are shown (n = 10–12 per group). Bars, 100 μm. (d, e) TNF-α and IL-1β in BAL were measured by ELISA. (f) The scheme shows that in response to IL-37 ligation, Sigirr disassociates from USP13, promoting Sigirr ubiquitination and degradation. USP13 plays an anti-inflammatory role through the stabilization of Sigirr. For all panels, the p-value was calculated by two-way ANOVA with *post hoc* Tukey's test *p < 0.05, **p < 0.01.

To investigate if the enhanced inflammation observed in *Usp13*^*−/−*^ mice is due to the dysregulation of Sigirr, we constructed a lentiviral vector encoding a fusion of Sigirr (LentiSigirr) and inoculated WT or *Usp13*^*−/−*^ mice with this construct or an empty control lentiviral vector (Lenticont) by intratracheal administration 5 d prior to intratracheal LPS injection. Expression of Sigirr by LentiSigirr infection in lung tissues was confirmed by Western blotting (Supplemental Fig. S7). The LentiSigirr transduction effectively dampened the enhanced LPS-induced lung inflammation phenotype observed in *Usp13*^*−/−*^ animals, with reduced cellular infiltration and neutrophils (Fig. 7a–c), TNF-α (Fig. 7d), and IL-1β (Fig. 7e). These data indicate that the augmentation of pro-inflammatory responses caused by USP13 depletion is in part due to the destabilization of Sigirr.

## 4 Discussion

IL-1R8/Sigirr has been shown to exhibit broad anti-inflammatory properties as its reduction augments inflammation and immune system-related disorders such as intestinal inflammation [2], allergy [31], and lung inflammation [14]. Understanding the molecular regulation of Sigirr protein expression may be helpful for identifying molecular targets that modulate Sigirr signaling. Here we identify USP13 as the first DUB that stabilizes Sigirr. We found that USP13 levels were reduced with corresponding decreases in Sigirr in response to endotoxin exposure in lung tissues from inflammatory murine models. Deletion of USP13 attenuated anti-inflammatory effects of Sigirr and increased the severity of lung inflammation in two murine models of lung injury. Conversely, ectopic expression of Sigirr effectively rescued USP13 knockout mice from LPS-induced inflammatory lung injury. This study suggests that Sigirr stability is normally maintained by USP13. Both are decreased in response to LPS or bacterial challenge, leading to more potent inflammatory responses.

Regulation of receptor stability is often mediated by post-translational modification with ubiquitin or other molecules and is central to cytokine and receptor signaling and biological effects. In recent years, ubiquitin biology has been demonstrated as critical to the regulation of multiple receptors and signal proteins. Until now, regulation of the stability of the anti-inflammatory Sigirr protein has not been described. The current study reveals that Sigirr is rapidly decreased after ligation by IL-37 as has been demonstrated for related cytokine receptors ST2L [26] after ligand IL-33 engagement. We now understand that Sigirr degradation is mediated by the attachment of K48-linked ubiquitin chains, which leads to Sigirr degradation in the proteasome. We identified the K163 lysine residue within the Sigirr intracellular domain as a ubiquitin acceptor site for this degradation; however, ubiquitination on K163 is not necessary for Sigirr internalization, suggesting that the modification of some other motif or other post-translational modification (such as phosphorylation) may be necessary for Sigirr internalization. Receptor internalization and degradation are uncoupled in other systems we have studied [28], and ubiquitination as well as phosphorylation are known molecular signals for receptor internalization [32]. Other Sigirr modifications that impact internalization, signaling, and stability will be areas of interest for future studies. Sigirr can also be constitutively degraded without ligand stimulation. In this study, we show that Sigirr is degraded in IL-37-dependent and -independent manners. Notably, we used a truncated form of IL-37 in this study. We will confirm the effects of other IL-37 isoforms on Sigirr degradation in future works.

Protein ubiquitination can be reversed by DUBs, which cleave ubiquitin or ubiquitin-like proteins from substrate proteins to prevent their degradation [19]. Our finding indicates that USP13 regulates Sigirr stability through the association and deubiquitination of Sigirr in both IL-37- dependent and independent pathways. In this study, we show that Sigirr abundance is reduced after LPS treatment in human and mouse cell and animal models. Though LPS has been shown to increase IL-37 mRNA stability in human cells [33], IL-37 is not present in mice. The decrease in Sigirr level we observe in mouse models may therefore be attributable to USP13 reduction rather than IL-37 signaling. USP13 targets multiple ubiquitinated proteins to modulate tumorigenesis through deubiquitination and regulation of PTEN [34], Myc [35], Beclin-1 [36], microphthalmia-associated transcription factor [37], and Siah2 [38]. USP13 also has been shown to regulate viral responses through the deubiquitination of STAT1 [39] and STING [40]. Our data indicate that USP13 exerts anti-inflammatory activity by stabilizing Sigirr. The inhibitory effect of USP13 on LPS signaling is dependent on its activity. *Usp13* deficient mice display increased severity of lung injury. While destabilization of other USP13 targets involved in inflammation such as STAT1, STING, or PTEN may also contribute to the pro-inflammatory effects we observe with *Usp13* knockout, none of these substrates is known to suppress inflammation as Sigirr does. Though most immune cells express Sigirr, it has been shown that M1 differentiated macrophages express more surface and total Sigirr compared with M2 macrophages and monocytes [41]. The molecular regulation of Sigirr degradation in different cell types needs more in-depth investigations. The expression of USP13 in M1 and M2 differentiated macrophages and monocytes needs to be studied in future works.

In summary, we propose a working model with USP13 as a positive regulator of Sigirr stability, and thus USP13 activity maintains anti-inflammatory IL-37-Sigirr activity with decreases in the abundance of both proteins during inflammation. USP13 deubiquitinase activity reverses Sigirr polyubiquitination and stabilizes the Sigirr protein to preserve anti-inflammatory signaling (Fig. 7g). Based on this information, stabilization of Sigirr could be achieved through enhancing USP13 activity, and this may be a practical strategy to block pulmonary inflammation.

## Appendix A Supplementary data

Supplementary material Image 1
