# Supplementary material for: The deubiquitinase USP13 stabilizes the anti-inflammatory receptor IL-1R8/Sigirr to suppress lung inflammation
Source: eBioMedicine. 2019 Jun 14;45:553–62. doi: 10.1016/j.ebiom.2019.06.011 (PMC6642080; doi:10.1016/j.ebiom.2019.06.011)
Supplement: Supplementary file 1 — Supplementary material [file mmc1.docx]

**Supplemental data**

**The dubiquitinase USP13 stabilizes the anti-inflammatory receptor IL-1R8/Sigirr to suppress lung inflammation**

**Lian Li^1,2^, Jianxin Wei^4^, Shuang Li^3^, Anastasia M. Jacko^4^, Nathaniel M. Weathington^4^, Rama K. Mallampalli^5^, Jing Zhao^1^, Yutong Zhao^1*^**

^1^Department of Physiology and Cell Biology, The Ohio State University, Columbus, Ohio, USA; ^2^Department of Respiration Medicine, Tianjin Medical University General Hospital, Tianjin, China; ^3^Department of Surgery, The first affiliated hospital of Dalian Medical University, Dalian, China; ^4^Department of Medicine, University of Pittsburgh School of Medicine, Pittsburgh, Pennsylvania, USA; ^5^Department of Medicine, The Ohio State University, Columbus, Ohio, USA

**
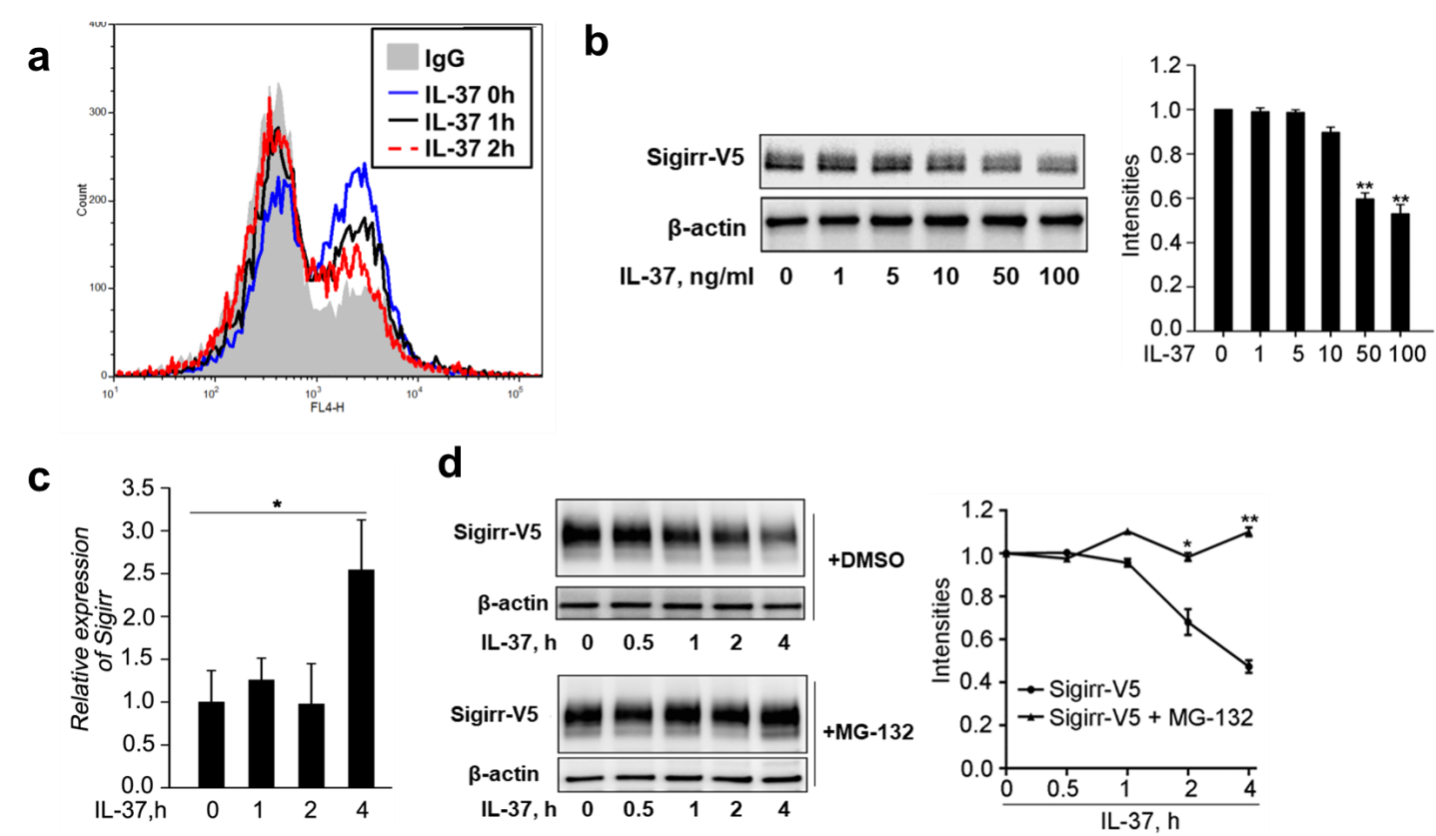
**

**Supplemental Figure S1. Sigirr is degraded in the proteasome. (a)** MLE12 cells were treated with IL-37 (100 ng/ml) for 0-2 h. Sigirr levels on the cell surface were measured by flow cytometry with an anti-Sigirr antibody. Shown is the representative image from three independent experiments. **(b)** MLE12 cells were transfected with plasmid encoding Sigirr-V5 for 48 h, and then treated with IL-37 in various concentrations. Cell lysates were analyzed by immunoblotting with antibodies to V5 and β-actin. Shown are the representative Western blots from three independent experiments. Intensities of Sigirr-V5 were evaluated by densitometric analysis with Image J software and overall p value was calculated by one-way ANOVA. Post hoc Tukey’s test was performed. All data are expressed as the mean ± SEM. *n*=3, ***p* < 0.01. **(c)** MLE12 cells were treated with IL-37 (100 ng/ml) for 0-4 h. qRT-PCR was used to analyze the relative mRNA expression of *Sigirr* and overall p value was calculated by two-way ANOVA. Post hoc Tukey’s test was performed. All data are expressed as the mean ± SEM. *n*=3, **p* < 0.05. **(d)** MLE12 cells were transfected with Sigirr-V5 plasmid for 48 h, and then treated with DMSO or MG-132 (20 µM) for 2 h prior to IL-37 treatment for indicated times. Cell lysates were analyzed by immunoblotting with antibodies to V5 and β-actin. Shown are the representative Western blots from three independent experiments. Intensities of Sigirr-V5 were evaluated by densitometric analysis with Image J software and overall p value was calculated by two-way ANOVA. Post hoc Tukey’s test was performed. All data are expressed as the mean ± SEM. *n*=3, **p* < 0.05; **p* < 0.01.


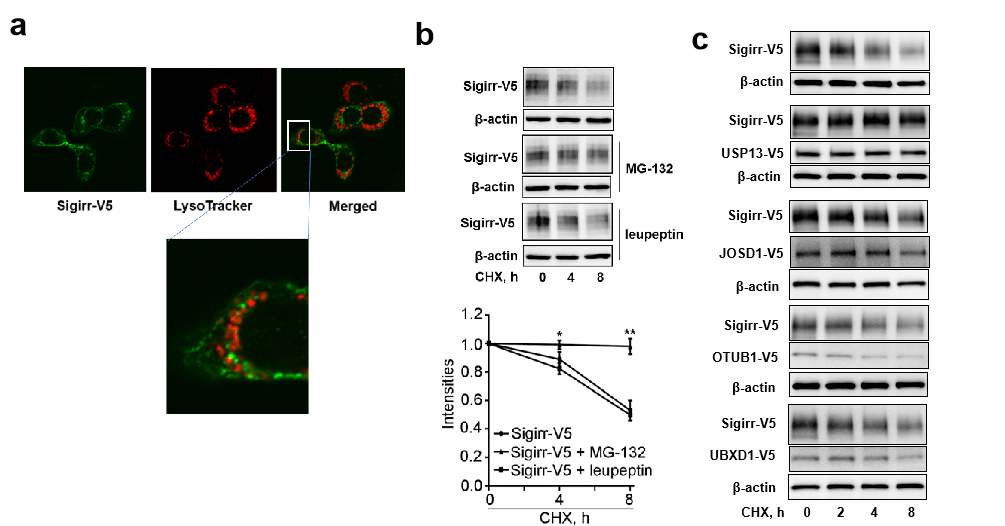


**Supplemental Figure S2. Sigirr degradation occurs in the proteasome and is dependent on ubiquitination. (a)** MLE12 cells were transfected with Sigirr-V5, and then treated with IL-37 for 1 h. The cells were immunostained with antibody to V5 tag (green) and lysotracker (red). Lack of yellow color indicates no lysosomal accumulation of Sigirr-V5. Shown are the representative images from three independent experiments. **(b)** MLE12 cells were transfected with plasmid encoding Sigirr-V5, and then treated with cycloheximide (CHX) alone or together with MG-132 or leupeptin for 0, 4, and 8 h. Cell lysates were analyzed by immunoblotting with antibodies against V5 tag and β-actin. Shown are the representative Western blots from three independent experiments. Intensities of Sigirr-V5 were evaluated by densitometric analysis with Image J software and overall p value was calculated by two-way ANOVA. Post hoc Tukey’s test was performed. All data are expressed as the mean ± SEM. *n*=3, **p* < 0.05; **p* < 0.01.


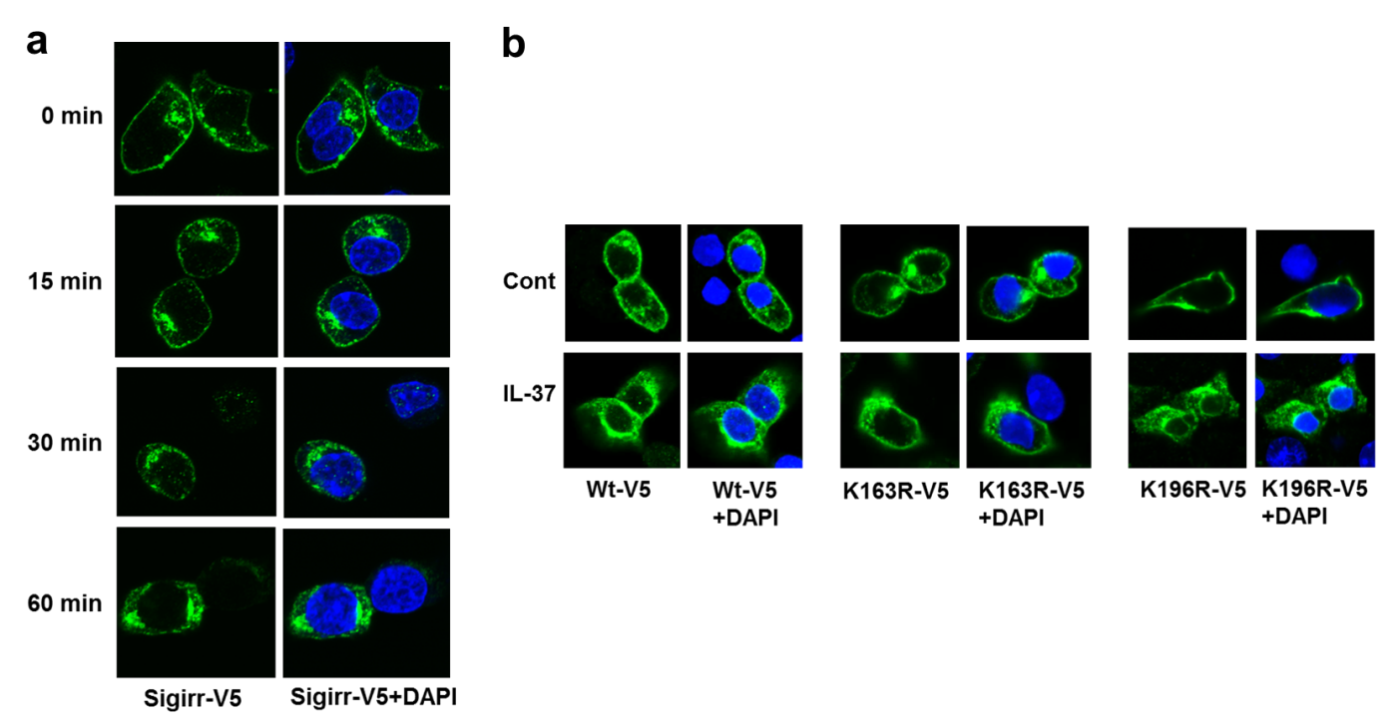


**Supplemental Figure S3. Ubiquitination on lysine 163 has no effect on Sigirr internalization. (a)** MLE12 cells were transfected with plasmid encoding Sigirr-V5 for 48 h, and then were treated with IL-37 (100 ng/ml) for 0-60 min. Cells were immunostained with an antibody against V5 tag (green). Nuclei were stained with DAPI (blue). **(b)** MLE12 cells were transfected with plasmids encoding Sigirr-V5, SigirrK163R-V5, or SigirrK196R-V5 for 48 h, and then were treated with IL-37 (100 ng/ml) for 60 min. Cells were immunostained with an antibody against V5 tag (green). Nuclei were stained with DAPI (blue). Shown are the representative images from three independent experiments.

**
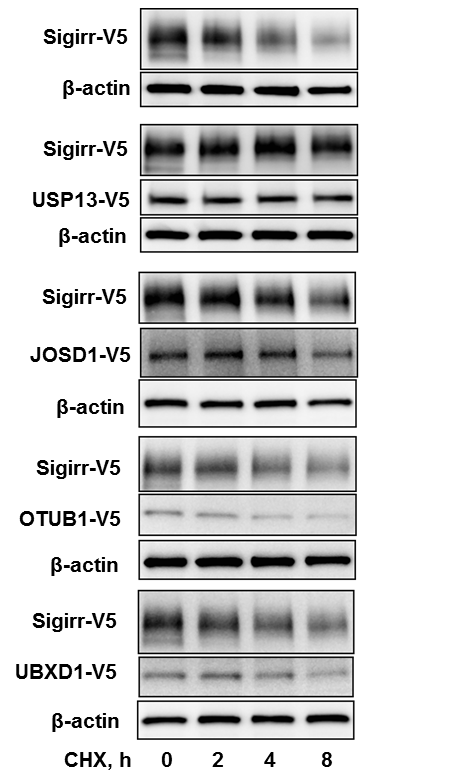
**

**Supplemental Figure S4. A screen to identify a DUB responsible for stabilizing Sigirr.** Plasmids encoding V5-tagged USP13, JOSD1, OTUB1, or UBXD1 were co-transfected with Sigirr-V5 into MLE12 cells, followed by CHX treatment. Cell lysates were analyzed by immunoblotting with antibodies to V5 tag and β-actin.


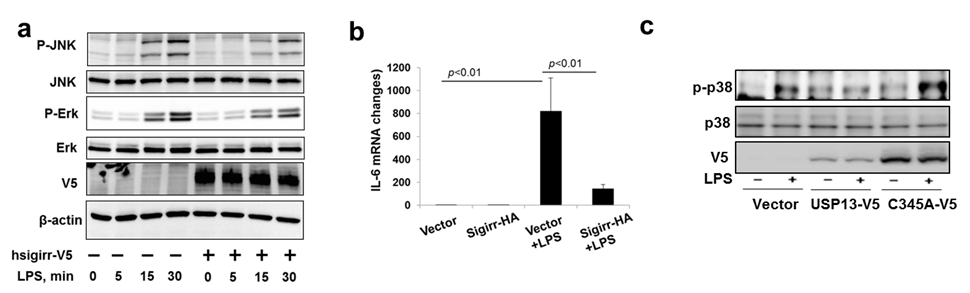


**Supplemental Figure S5. Overexpression of Sigirr attenuates LPS/TLR4 signaling. (a)** Cell lysates were analyzed by Western blotting with indicated antibodies. Shown are the representative Western blots from three independent experiments. (b) RAW264.7 cells were transfected with plasmid encoding Sigirr-V5 for 48 h, and then were treated with LPS (0.1 µg/ml) for 6 h. IL-6 mRNA levels (normalized to GAPDH mRNA levels) were measured by qRT-PCR and overall p value was calculated by two-way ANOVA. Post hoc Tukey’s test was performed. Shown are mRNA changes compared to vector alone. *n*=3. Error bars indicate mean ± SEM. (c) 293TLR4 cells were transfected with plasmids encoding control empty vector, USP13-V5 or USP13C345A-V5, and then cell were treated with LPS (0.1 µg/ml) for 30 min. Cell lysates were analyzed by immunoblotting with indicated antibodies.


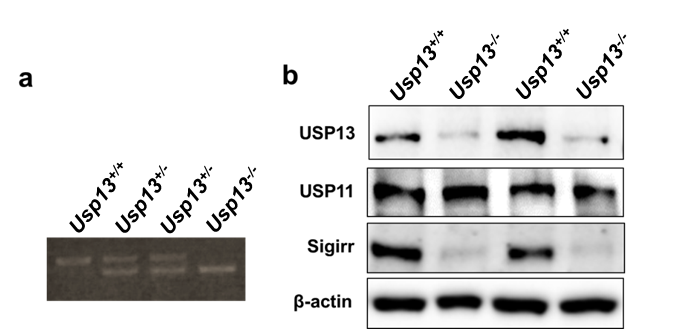


**b**

**a**


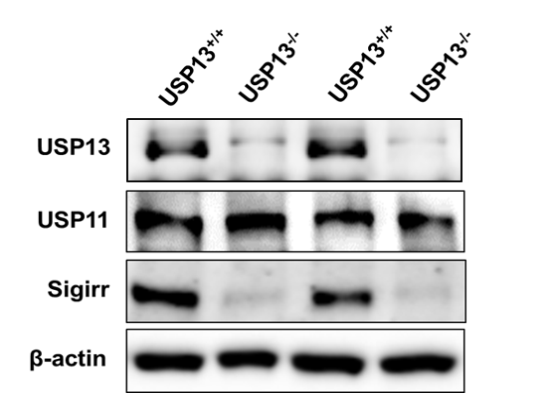


**Supplemental Figure S6. Sigirr levels are reduced in *Usp13^-/-^* mice. (a)** Genotyping PCR identify *Usp13^+/+^*, *Usp13^+/-^*, *Usp13^-/-^* mice. Upper band only, wild type; two bands, heterozygous; lower band only, homozygous. Shown are the representative PCR images. **(b)** Lung tissue lysates were analyzed by immunoblotting with antibodies against USP13, USP11, Sigirr, and β-actin. Shown are the representative Western blots from *n*=3.


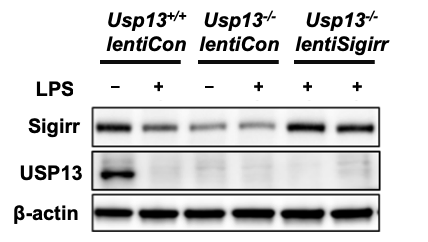


**Supplemental Figure S7. Overexpression of Sigirr by a lentiviral vector delivery system.** WT and *Usp13^-/-^* mice were subjected to intratracheal injection of control lentivirus (LentiCon) or lentivirus encoding Sigirr (lentiSigirr) for 5 d, and then intratracheal administration of LPS (2 mg per kg body weigh) for 24 h. Lung tissue lysates were analyzed by Western blotting with indicated antibody. Shown are representative blots (*n*=3).
